# The Ykt6–Snap29–Syx13 SNARE complex promotes crinophagy via secretory granule fusion with Lamp1 carrier vesicles

**DOI:** 10.1038/s41598-024-53607-x

**Published:** 2024-02-08

**Authors:** Győző Szenci, Gábor Glatz, Szabolcs Takáts, Gábor Juhász

**Affiliations:** 1 Department of Anatomy, Cell and Developmental Biology, Eötvös Loránd University, Budapest, 1117 Hungary; 2 Doctoral School of Biology, Institute of Biology, Eötvös Loránd University, Budapest, 1117 Hungary; 3 Institute of Genetics, HUN-REN Biological Research Centre Szeged, Szeged, 6726 Hungary

**Keywords:** SNARE, Membrane proteins

## Abstract

In the Drosophila larval salivary gland, developmentally programmed fusions between lysosomes and secretory granules (SGs) and their subsequent acidification promote the maturation of SGs that are secreted shortly before puparium formation. Subsequently, ongoing fusions between non-secreted SGs and lysosomes give rise to degradative crinosomes, where the superfluous secretory material is degraded. Lysosomal fusions control both the quality and quantity of SGs, however, its molecular mechanism is incompletely characterized. Here we identify the R-SNARE Ykt6 as a novel regulator of crinosome formation, but not the acidification of maturing SGs. We show that Ykt6 localizes to Lamp1+ carrier vesicles, and forms a SNARE complex with Syntaxin 13 and Snap29 to mediate fusion with SGs. These Lamp1 carriers represent a distinct vesicle population that are functionally different from canonical Arl8+, Cathepsin L+ lysosomes, which also fuse with maturing SGs but are controlled by another SNARE complex composed of Syntaxin 13, Snap29 and Vamp7. Ykt6- and Vamp7-mediated vesicle fusions also determine the fate of SGs, as loss of either of these SNAREs prevents crinosomes from acquiring endosomal PI3P. Our results highlight that fusion events between SGs and different lysosome-related vesicle populations are critical for fine regulation of the maturation and crinophagic degradation of SGs.

## Introduction

Professional secretory cells produce large amounts of secretory material (hormones, neuropeptides, digestive enzymes, mucin, etc.) and store them in secretory granules (SGs) until a secretagogue elicits their bulk exocytosis. These cells usually produce more secretory material than is released by exocytosis^1–3^ to provide a sufficient pool of available SGs^4^. Secretory cells continuously turn over the excess SGs by crinophagy, a specialized form of autophagy to maintain a constant releasable pool of SGs^2,5–7^. Following this route, abnormal or obsolete SGs may also be subject to crinophagic degradation^3,7,8^. In addition to degradative crinophagy, SG-lysosome fusions may also contribute to the complex maturation process of SGs and thereby determine their controlled release by exocytosis. During crinophagy, SGs directly fuse with lysosomes that gives rise to degradative crinosomes^9^.

Easy genetic manipulation and highly conserved molecular mechanisms made *Drosophila* a powerful in-vivo model for deciphering the molecular regulation of the regulated secretory pathway and crinophagy. Salivary gland cells produce^10,11^ and secrete^12^ high amounts of Sgs (Salivary gland secretion)/glue proteins in response to peaks of the molting hormone ecdysone^10–12^. The released glue is then expelled from the lumen to anchor the metamorphosing prepupae to solid surfaces^12^. The nascent glue SGs emanate from the TGN^13,14^, increase in size by homotypic fusions^15,16^, and then undergo a complex maturation process during which SGs fuse with lysosomes. This promotes the acidification and profound reorganization of the inner content of SGs^3,17–19^, preparing them for secretion^17,19,20^. Excess or abnormal glue can be also degraded by crinophagy, through fusion of non-secreted SGs and lysosomes^3,7,8,19,21,22^. Taken together, crosstalk and fusion between SGs and the endolysosomal compartment is critical both for SG maturation and crinosome formation, however, the molecular mechanism of these processes is still incompletely understood^3,18,19,21–23^.

By enabling direct fusion between SGs and lysosomes, crinophagy differs mechanistically from the canonical main autophagic pathway, which mediates the degradation of cytosolic material through autophagosome formation and their subsequent fusion with lysosomes. Accordingly, genes that are required for autophagosome formation proved dispensable to crinophagy^3,24,25^, while SG-lysosome fusion itself relies on a similar molecular machinery acting in fusions between autophagosomes and lysosomes^3,5,21,22^. The machinery mediating autophagosome-lysosome fusion is well characterized both in Drosophila and humans by now. Critical components include Rab2, Rab7, and Arl8 small GTPases that also contribute to defining membrane identity^21,26–29^, homotypic fusion and vacuole protein sorting (HOPS) tethering complex^30,31^, and a soluble N-ethylmaleimide-sensitive factor attachment protein receptor (SNARE) complex (SNAREpin) that executes the fusion. Based on biochemical properties, functional SNAREpins assemble from three Q-(Q_abc_) and one R-SNARE domains^32^. The first discovered SNAREpin that mediates autophagosome-lysosome fusion is composed of Syntaxin 17, Snap29 and Vamp7/8^30,31,33,34^. Recently another R-SNARE: Ykt6 was also discovered to also have a role in the process, either as an R-SNARE potentially substituting for Vamp7^35^ or interacting with Syntaxin 7 and Snap29 to form an alternative SNAREpin^36,37^. Interestingly, Drosophila crinophagic fusion of glue SGs and lysosomes depends on highly similar machinery, composed of Rab2, Rab7, Arl8, HOPS and a Syntaxin 13, Snap29 and Vamp7 SNAREpin^3,21,22^. The similarity of the molecular machinery regulating these lysosomal fusions raised the possibility that Ykt6 may also regulate SG-lysosome fusions and crinophagy.

Ykt6 is a highly conserved R-SNARE that consists of an N-terminal longin domain (LD), an R-SNARE domain, and a conserved C-terminal lipidation motif with the amino acid sequence CCAIM. The latter is critical for membrane association^38–42^ because Ykt6, unlike other R-SNAREs, lacks a canonical transmembrane domain. Moreover, the lipid anchors can hide reversibly in the hydrophobic groove of the protein, which enables Ykt6 to leave membranes and form a cytosolic pool^38–41^. This way, it can be rapidly incorporated into various intracellular membranes on demand and form a complex with compartment-specific Q-SNAREs to promote vesicle fusion. Membrane-associated Ykt6 regulates the anterograde ER to Golgi^43,44^, the intra-Golgi^45–47^, retrograde directed Golgi to ER, and endosome to TGN transports^48^, and the release of constitutive secretory carriers^49^ or exosomes^50,51^ along the secretory pathway. In addition, it also promotes biosynthetic transport to the yeast vacuole and lysosomes in animal cells^42,52^, endosomal recycling^53^, and macroautophagic degradation^35–37,54,55^. However, the role of Ykt6 in SG-lysosome fusion and crinophagy remained unknown.

Here, we show that Ykt6 forms a canonical SNAREpin with Syntaxin 13 and Snap29, which is—similarly to the already known Syntaxin 13, Snap29, Vamp7 SNAREpin—critical for crinophagic degradation. We also demonstrate that Ykt6 localizes to small Lamp1+ (carrier) vesicles and mediates their fusion with SGs, while Vamp7 regulates the fusion of SGs and Arl8+ lysosomes. In summary, we provide evidence that SG maturation preceding exocytosis and crinophagy requires a series of fusions between SGs and two separate lysosome-related vesicle subpopulations, which are governed by different SNAREpins/SNARE complexes.

## Results

### Ykt6 is required for crinophagic degradation

**Figure 1:**
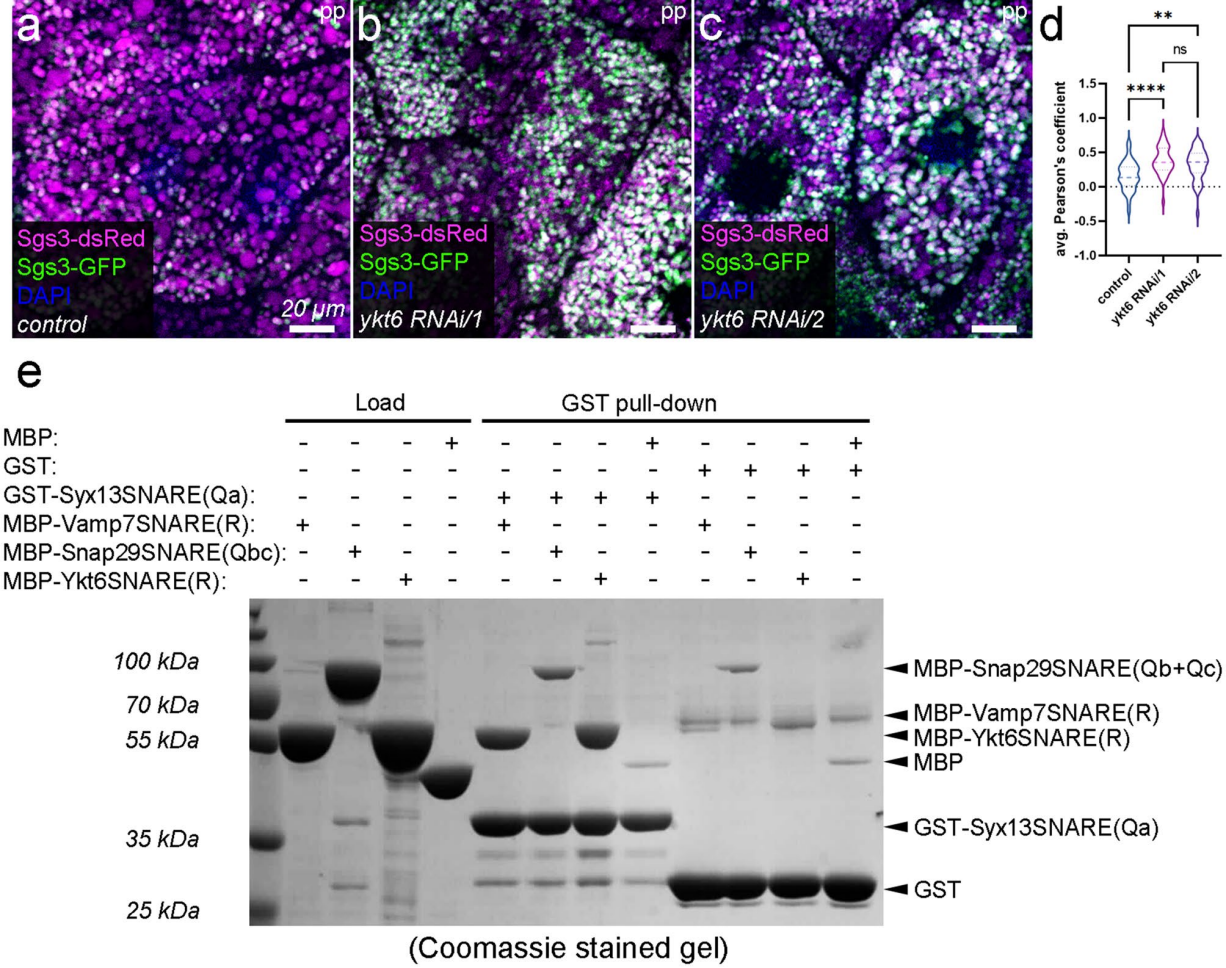
Ykt6 forms a SNARE complex with Syntaxin 13 Q_a_- and SNAP29 Q_bc_ SNAREs to regulate the crinophagic degradation of SGs. (**a**) In the salivary gland cells from control prepupae (pp), most Sgs3-GFP signals are quenched due to the fusion of SGs with acidic lysosomes, resulting in the appearance of dsRed-only degradative crinosomes. (**b**–**d**) In contrast, RNAi-mediated silencing of *ykt6* by two independent RNAi transgenes leads to defective crinophagic degradation based on retained Sgs3-GFP signal. (**d**) Quantification of the overlap between Sgs3-GFP and Sgs3-dsRed markers from (**a**–**c**), n = 40 cells from 8 different larvae. Dashed lines mark the median and the dotted lines are the upper and lower quartiles of violin plots. **p < 0.01, ****p < 0.0001, ns p > 0.05. (**e**) GST pull-down experiment with N-terminally GST- or MBP-tagged recombinant SNARE domains purified from *E. coli*. GST alone served as a negative control. The immobilized GST-Syntaxin13 Q_a_ bait strongly interacts with MBP-tagged Snap29 Q_bc_-, and Vamp7 or Ykt6 R-SNARE prey motifs. This suggests that Ykt6 can assemble with the Syntaxin 13 Q_a_ and Snap29 Q_bc_ SNARE proteins to form a functional SNAREpin, similar to the previously identified crinophagic Syntaxin 13, Snap29 and Vamp7 SNAREpin.

The crinophagic SG-lysosome and the conventional autophagosome-lysosome fusions share key regulators^7^. Since the possible role of Ykt6 as an alternative R-SNARE in SG-lysosome fusion has not been investigated so far, we analyzed the putative role of Ykt6 in crinophagic SG degradation. We carried out loss of function experiments by silencing *ykt6* in prepupal (pp) salivary gland cells and assayed its effect on crinophagic flux. The acidification and lysosomal degradation of glue SGs can be monitored by simultaneous expression of the N-terminal GFP- and dsRed-tagged Sgs3 glue protein in the larval salivary glands. The differently labeled Sgs3 reporters are both in the lumen of forming SG, so these are initially positive for both fluorophores. Their fusion with acidic lysosomes results in quenching the GFP signal due to the acidic environment. Therefore, at the time of puparium formation, most of the SGs that are not secreted remain positive only for dsRed^3^. To investigate the consequence of Ykt6 loss, we used time-controlled RNA interference (RNAi)-mediated knock-down of the protein^53^, to circumvent its possible undesirable effect on the biogenesis of SGs. In control cells, most of the SGs appear dsRed-only as the Sgs3-GFP signal is quenched in acidic milieu (Fig. 1a,d). In contrast, many dsRed and GFP double-positive SGs remain in *ykt6* silenced cells (Fig. 1b–d), indicating defective crinophagic SG degradation. This phenotype resembled the absence of the previously described crinophagic SNAREs, Syx13, Snap29 and Vamp7^3^. Thus, Ykt6 may mediate crinophagic SG-lysosome fusion in a similar way to Vamp7.

### The R-SNARE Ykt6 forms a SNARE complex with Syntaxin13 Q_a_ and Snap29 Q_bc_ SNAREs

To test the ability of Ykt6 to form a functional SNAREpin, we examined its interactions with the previously identified crinophagic Q-SNAREs Syntaxin 13 and Snap29^3^ by performing a GST pull-down assay with N-terminally GST- or MBP-tagged SNARE domains. GST-Syntaxin 13 Q_a_ (bait) was immobilized on glutathione beads, and we observed its strong interaction with the MBP-tagged Snap29 Q_bc_ and Vamp7 as well as Ykt6 R-SNARE (prey) domains (Fig. 1e). Importantly, the binding of recombinant Ykt6 to Syx13/Snap29 was much stronger than to the Syx17/Snap29 autophagic SNARE complex^35^. This finding indicates that Ykt6 regulates crinophagy by forming a SNAREpin with the crinophagic Q-SNAREs.

### Acidification of maturing SGs is regulated differently by Ykt6 and Vamp7

**Figure 2:**
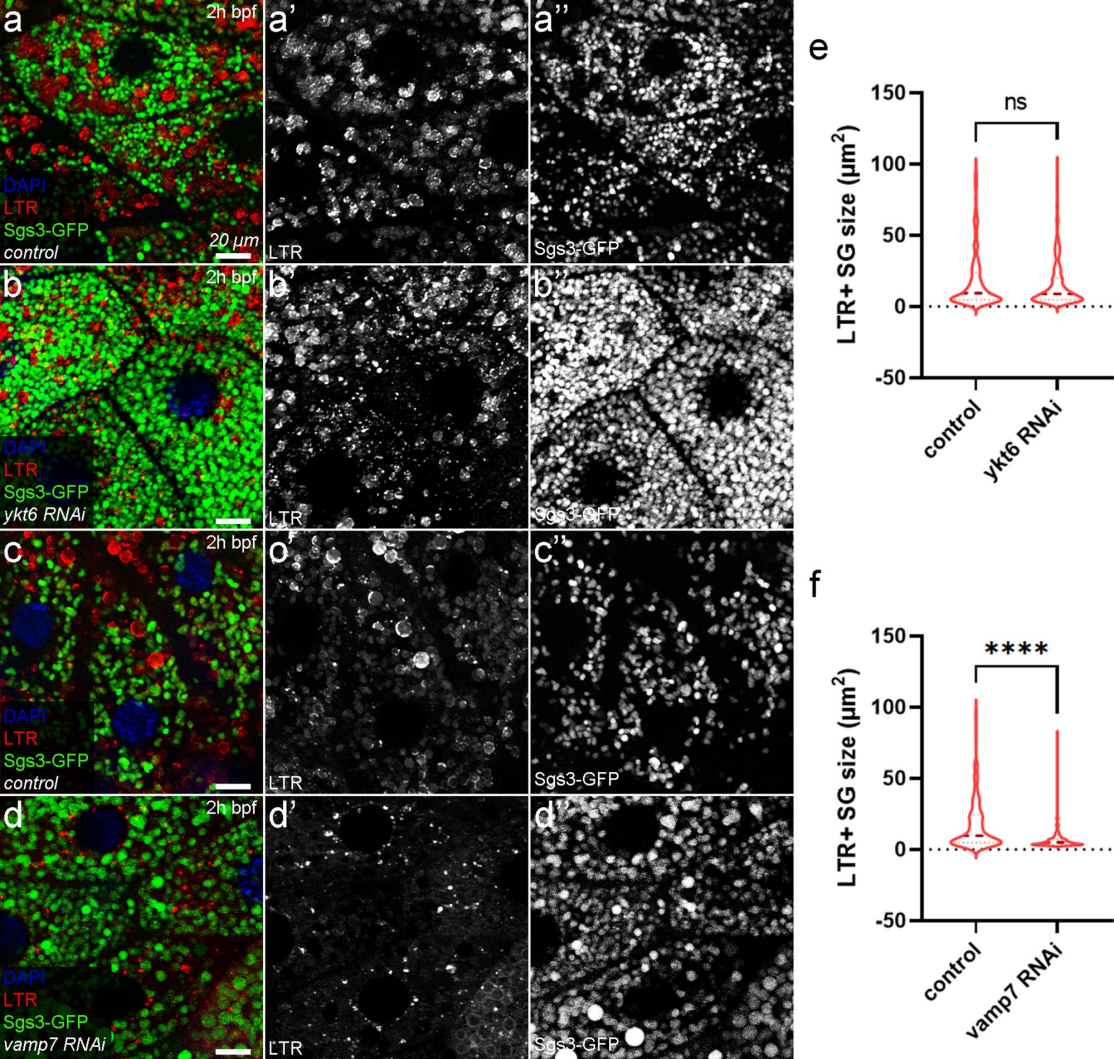
Ykt6 and Vamp7 R-SNAREs differentially regulate the acidification of maturing secretory granules. (**a**) In control salivary gland cells, maturing SGs become positive for LysoTracker Red, and their Sgs3-GFP fluorescence is quenched due to the acidic milieu. Loss of either of the two R-SNAREs impacted the proper acidification of maturing SGs differently. The absence of *ykt6* (**b**) did not have a statistically significant effect on the size of LTR+ structures. In contrast, the loss of *vamp7* (**d**) strongly inhibited the acidification of maturing SGs compared to the corresponding control (**c**), resulting in the accumulation of smaller (presumably fusion incompetent) LTR+ lysosomes. (**e**,**f**) Quantification of the size of LTR+ structures shown in (**a**–**d**), n = 250 LTR+ structures from 5 cells of 5 different larvae, ****p < 0.0001, ns p > 0.05.

In addition to degradative crinosomes (at the prepupal stage—pp), ecdysone-induced progressive acidification also accompanies the maturation of SGs (2 h before puparium formation—bpf), and is important for the remodeling of the inner structure of SGs and prepares them for exocytosis (priming)^3,17–19,56,57^. Although both Vamp7 and Ykt6 proved to be required for the acidification of degradative crinosomes at the prepupal (pp) stage, it was still elusive whether these two R-SNAREs are also equally required for proper acidification of maturing SGs before their release. To assay this, we stained the genomic Sgs3-GFP expressing salivary glands of 2 h bpf larvae with LysoTracker Red (LTR), a vital dye that labels acidic structures (Fig. 2). In control cells around the time of robust secretion, mature SGs had already lost their GFP fluorescence, accompanied by a parallel accumulation of large LTR+ vesicles (Fig. 2a,c). In the absence of *ykt6*, no statistically significant difference was observed in the size of LTR+ structures compared to the control cells (Fig. 2a,b,e) even though the Glue-GFP signal was already higher compared to the control (Fig. 2a,b), similar to the crinophagic flux experiments (Fig. 1a–c). In contrast, the lack of Vamp7 strongly reduced the size of LTR+ acidic structures compared to the respective control (Fig. 2c,d,f). These findings raised the possibility that the two R-SNAREs required for crinophagy play different roles in the acidification and maturation of Glue SGs.

### Ykt6 and Vamp7 differently regulate the fusion of Arl8+ lysosomes with SGs

**Figure 3:**
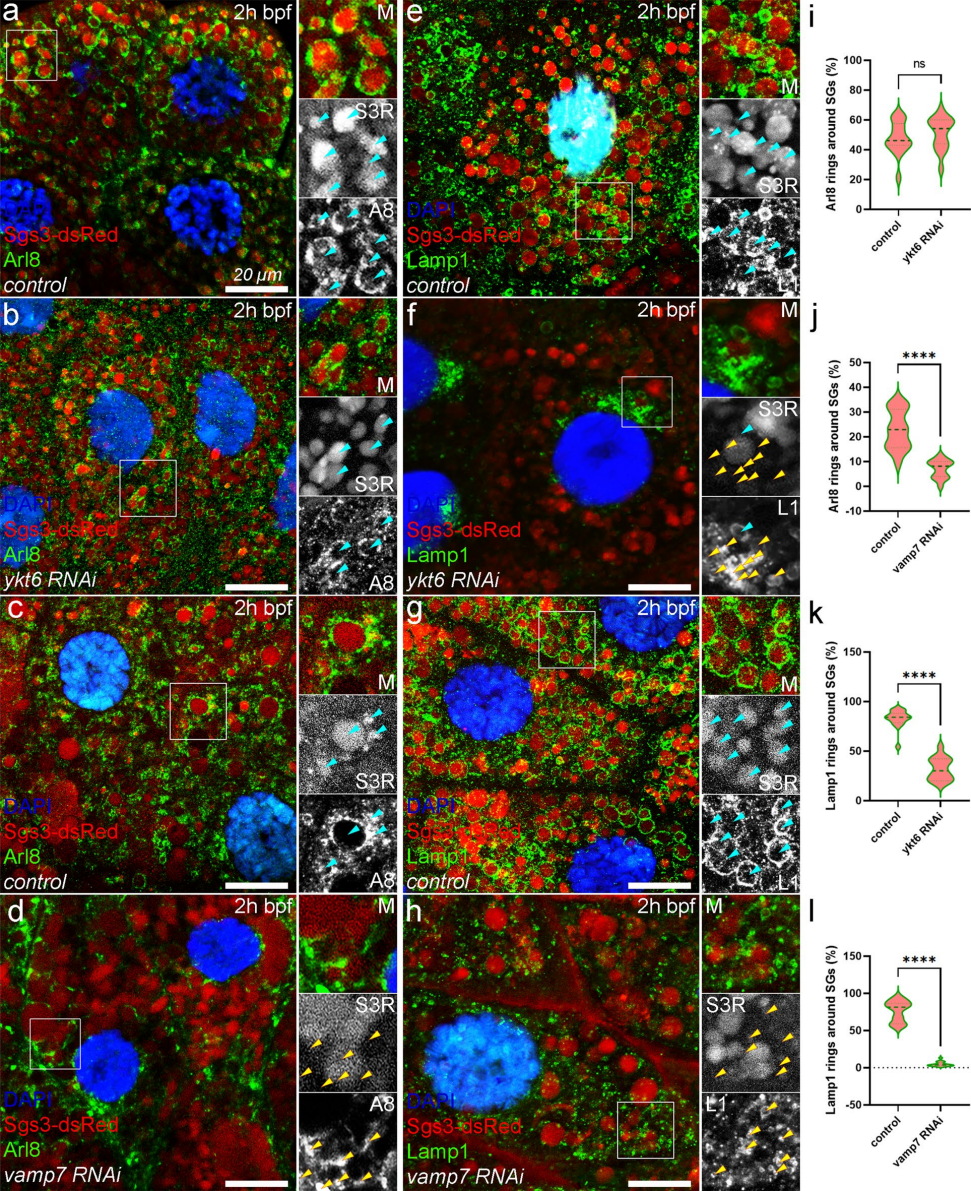
Ykt6 is required for fusion of SGs with Lamp1+ but not Arl8+ vesicles, while Vamp7 is required for both. (**a**,**c**) In control larvae, maturing SGs fuse with Arl8+ lysosomes leading to the ring-like appearance of Arl8 around the Sgs3-dsRed+ SGs (turquoise arrowheads). Knockdown of *ykt6* (**b**) does not affect these types of lysosomal fusions as Arl8 rings still appear around most SGs. (**c**,**d**) However, these fusions are inhibited in *vamp7* silenced salivary glands (**d**) compared to the control (**c**), causing aggregation of the fusion incompetent Arl8+ lysosomes (yellow arrowheads) between the SGs (**d**). (**e–h**) Fusion of Lamp1+ vesicles supports the maturation of SGs and Lamp1 forms rings around SGs in control cells in a similar fashion to Arl8 (**e**). *Ykt6* deficiency (**f**) strongly inhibits the fusion of SGs with Lamp1+ lysosomes/carrier vesicles, leading to the clustering of unfused dLamp1+ vesicles between SGs. (**g**,**h**) Silencing of *vamp7* also inhibits these kinds of fusions, resulting in the accumulation of small-size Lamp1+ lysosomes around SGs that are unable to fuse (**h**), unlike in the corresponding control (**g**). (**i**–**l**) Quantification of the data shown in (**a**–**h**), n = 875 (**a**), n = 772 (**b**), n = 956 (**c**), n = 611 (**d**) Arl8+ structures and n = 513 (**e**), n = 724 (**f**), n = 516 (**g**), n = 1186 (**h**) Lamp1+ structures from 3 cells of 5 different larvae, ****p < 0.0001, ns p > 0.05. Insets show the outlined areas magnified (×2) and split into channels in panels (**a**–**h**).

The maturation and crinophagic decomposition of glue SGs both rely on a series of fusion events between SGs and lysosomes or endosomes^3,8,18,19,21,22^. Since Vamp7 and Ykt6 differentially affected SG maturation/acidification, we supposed that these R-SNAREs may mediate the fusion of SGs with different components of the endo-lysosomal compartment. Therefore, we tested the colocalization between glue granules and different endo-lysosomal markers in *ykt6* RNAi or *vamp7* RNAi salivary glands, respectively. Arl8 is a small GTPase highly specific for lysosomes and it is necessary for direct fusion of Lamp1+ lysosomes and glue SGs^21^. In control cells, endogenous Arl8 forms rings around Sgs3-dsRed+ SGs (Fig. 3a,c) that indicates successful fusions between Arl8+ lysosomes and maturing SGs. The absence of *ykt6* does not interfere with fusion of Arl8+ lysosomes, as Arl8 still forms rings around SGs (Fig. 3b,i). In contrast, silencing of *vamp7* strongly inhibits the formation of rings around SGs: instead, Arl8 labels small vesicle aggregates (Fig. 3d,j). This reflects a strong defect in Arl8+ lysosome-SG fusions (Fig. 3c,d,j) and suggests that this fusion event is mediated by the SNAREpin containing Vamp7.

### Ykt6 is involved in the fusion of Lamp1+ vesicles with SGs

Lamp1 is a highly glycosylated transmembrane protein, an essential component of the lysosomal membranes^58^. Although Lamp1 is often used as a lysosome marker, it is also present on a broader spectrum of vesicles belonging to the endo-lysosomal compartment^27,59^. Similar to our observations with Arl8, endogenous Lamp1 also forms rings along the perimeter of maturing Sgs3-dsRed SGs in control cells 2 h bpf (Fig. 3e,g). However, the formation of these rings was strongly perturbed both in *ykt6* (Fig. 3f) and *vamp7* (Fig. 3h) silenced salivary gland cells (Fig. 3e–h,k,l). Thus, these lysosome markers are delivered to maturing SGs through independent fusion events governed by different R-SNAREs. Our data point to the involvement of a heterogeneous population of Arl8+ and Lamp1+ lysosomes and related vesicles in maturation and crinophagic degradation of glue SGs.

### Ykt6 and Vamp7 are required for endosomal fusions of SGs following secretion

**Figure 4:**
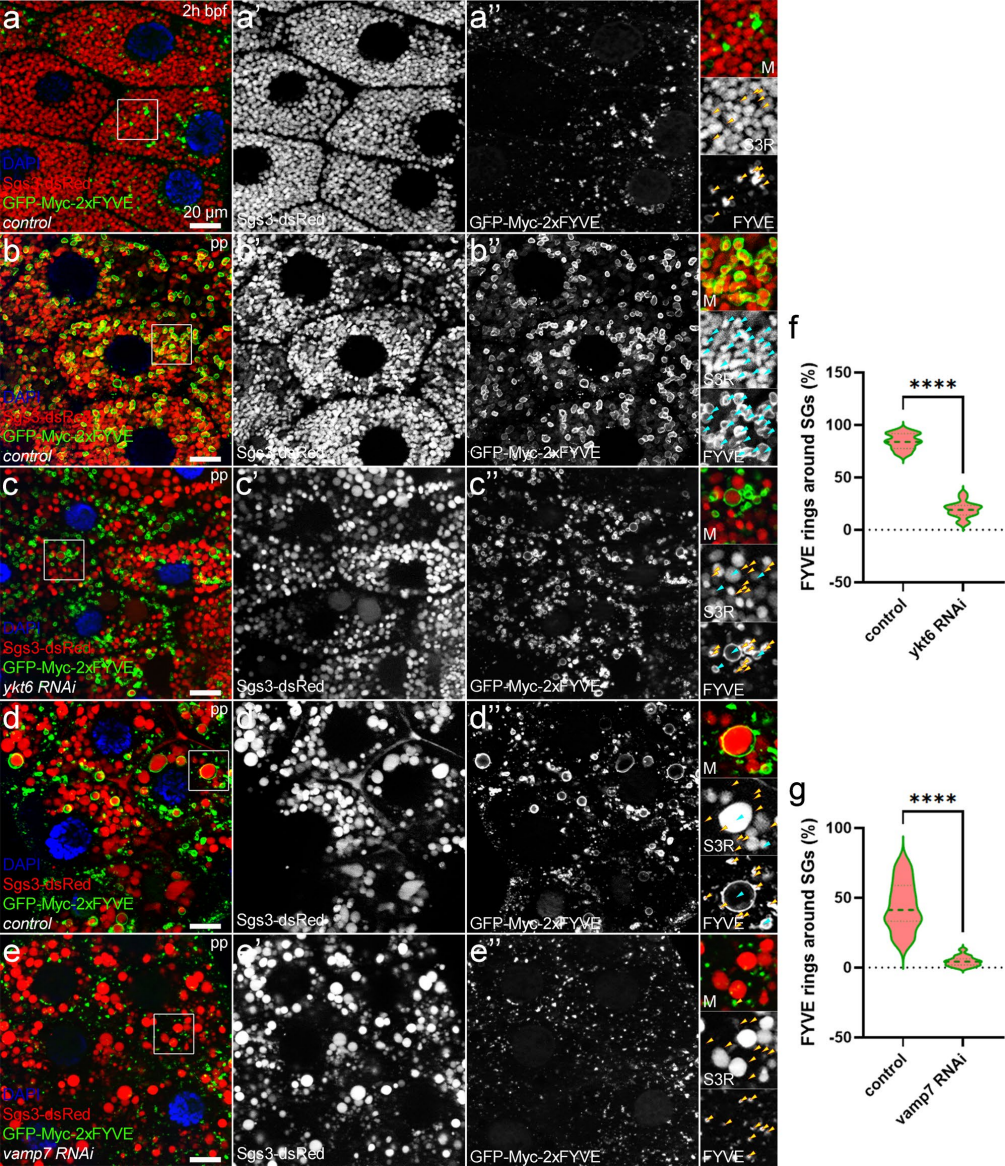
Vamp7 and Ykt6 mediated fusions are both required for PI3P positivity of residual SGs after secretion. Parallel with the onset of the massive release of SGs, PI3P+ endosomes are formed to restore the balance between the apical and basolateral membranes. These endosomes are visualized by the GFP-Myc-2xFYVE reporter and appear as small aggregates or rings among the SGs at this stage (**a**). Later on, these endosomes subsequently fuse with the SGs at the prepupal (pp) stage in control cells, forming rings around the SGs (turquoise arrowheads), promoting the transformation of mature SGs (**b**). In the absence of *ykt6* (**c**), GFP-FYVE+ endosomes form clusters rather than rings around SGs. (**d**–**e**) Compared to their respective control (**d**), *vamp7* silenced cells (**e**) also exhibit a failure in fusion of GFP-FYVE+ endosomes with mature SGs. (**f**,**g**) Quantification of the data shown in panels (**b**–**e**), n = 1205 (**b**), n = 1408 (**c**), n = 606 (**d**), n = 1154 (**e**) GFP-FYVE+ structures from 3 cells of 5 different larvae, ****p < 0.0001. Insets show the outlined areas magnified (×2) and split into channels in panels (**a**–**e**).

It was previously described that the maturation of SGs requires contribution from the endosomal system^60–63^, but it remains unclear whether the fusion of endosomes with SGs is important for crinosome formation. To explore this, we labeled endosomes harboring phosphatidylinositol 3-phosphate (PI3P) with GFP-Myc-2xFYVE probe specific for PI3P and tested its overlap with Glue-dsRed. We observed that GFP-FYVE marks usually small endosome clusters among the SGs at 2 h bpf (Fig. 4a.)^60–62^. Later on, non-secreted SGs trapped in cytosol are most likely removed by crinophagy by transforming into crinosomes^3,19^ which appear as large Sgs3-dsRed+ granules encircled by GFP-FYVE+ membranes (Fig. 4b,d). This suggests that crinosomes receive extensive membrane input from PI3P-positive endosomes. However, it remained unclear whether this endosomal input was dependent on the preceding fusion events between lysosomes and maturing SGs. We observed that in the lack of Ykt6, these PI3P+ endosomes are clustered between SGs, rather than forming a ring around them (Fig. 4b,c,f). Similarly, the absence of Vamp7 strongly inhibited the fusion of PI3P+ endosomes and SGs compared to the respective control (Fig. 4d,e,g). These results show that the Ykt6- and Vamp7-mediated lysosomal fusions determine the subsequent fate and fusion capacity of the SGs because they fail to fuse with PI3P+ endosomes in the absence of either SNARE.

### Ykt6 localizes to small Lamp1+ vesicles

**Figure 5:**
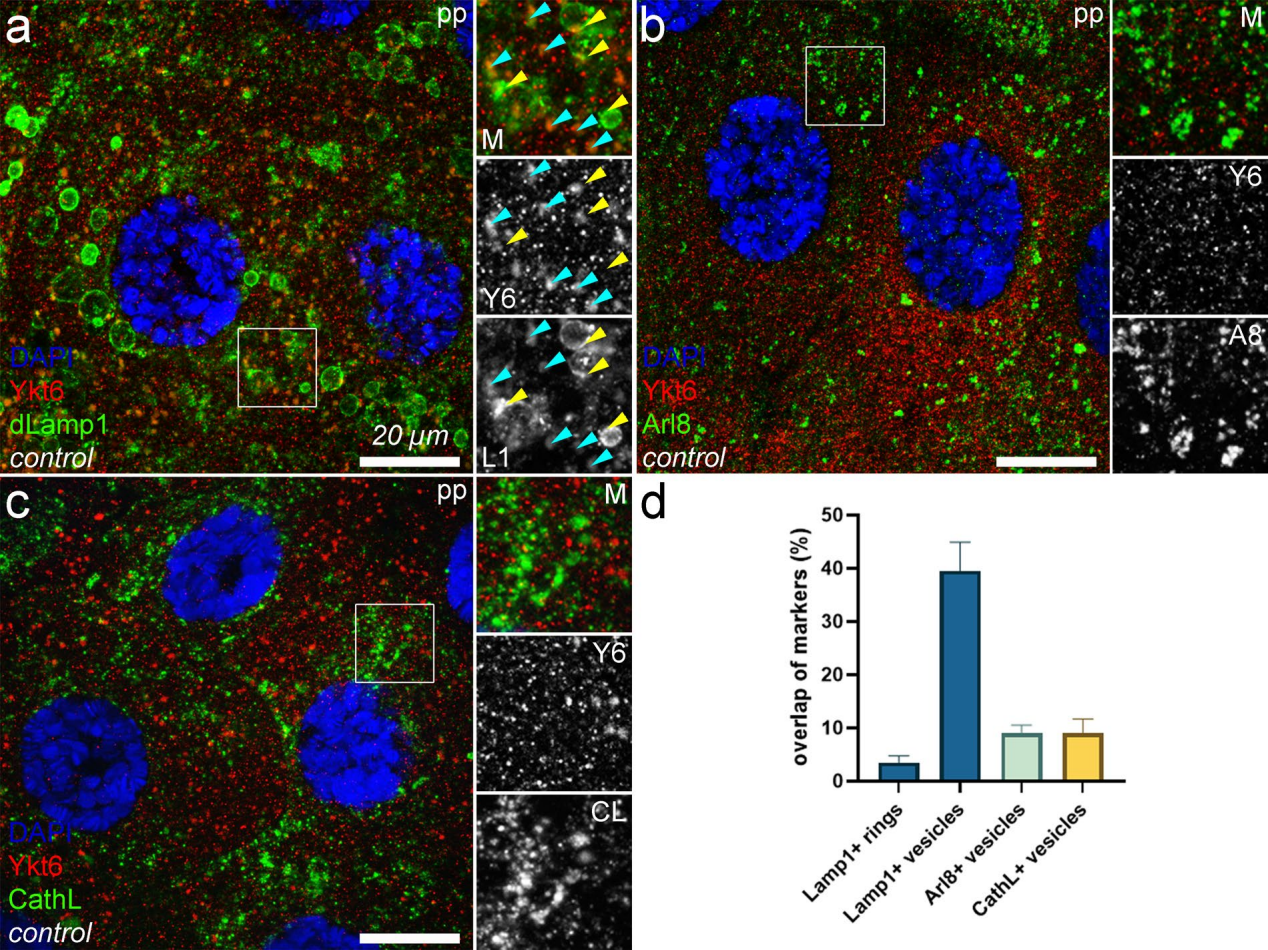
Ykt6 localizes to small Lamp1+ (carrier) vesicles. (**a**–**c**) Ykt6 localizes to small punctate structures that are evenly distributed in cells. (**a**) These vesicles often overlap with small Lamp1+ vesicles (turquoise arrowheads) while the larger Lamp1+ rings (yellow arrowheads) that form around maturing SGs are devoid of Ykt6. Ykt6 does not overlap with established lysosomal markers Arl8 (**b**) and Cathepsin L (**c**), consistent with the different roles of Vamp7 and Ykt6 in SG maturation-promoting lysosomal fusions. (**d**) Quantification of the overlap between markers in (**a**–**c**), n = 200 structures from 5 cells of 5 different larvae. Error bars mark ± SEMs. Insets show the outlined areas magnified (×2) and split into channels in panels (**a**–**c**).

Since we found that Ykt6 and Vamp7 mediate the fusion of maturing SGs with different lysosome-related vesicle populations, we also aimed to elucidate the subcellular localization of Ykt6. By carrying out immunolabeling with antibodies specific for Ykt6 and various lysosomal markers, we found that endogenous Ykt6 shows a punctate pattern which overlaps significantly with small Lamp1+ vesicles, while it is absent from the large Lamp1+ rings that presumably formed around maturing SGs (Fig. 5a,d). Ykt6 does not colocalize with other lysosome markers, such as Arl8 (Fig. 5b,d) or the lysosomal hydrolase Cathepsin L (Fig. 5c,d). These data are in line with our results that Ykt6 is mainly involved in the fusion of SGs and Lamp1+ vesicles, but not Arl8+ lysosomes (Fig. 3).

### Ykt6 does not affect the localization of Vamp7

**Figure 6:**
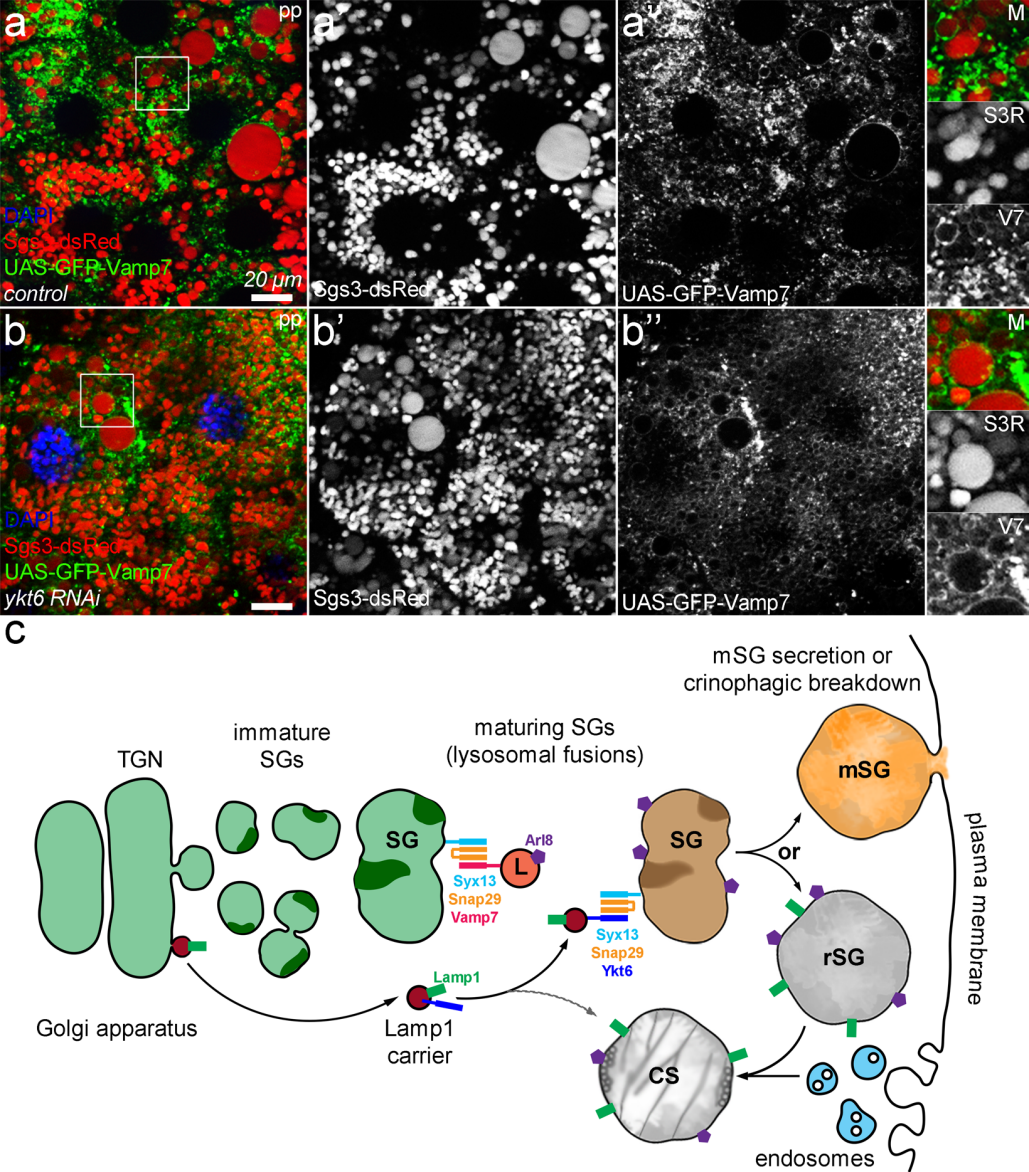
The absence of Ykt6 does not affect the localization of the other crinophagic R-SNARE, Vamp7. (**a**) In control prepupal salivary gland cells, GFP-Vamp7 forms rings around mature SGs and is also found on smaller structures that are presumably lysosomes or endosomes. (**b**) The absence of ykt6 does not affect the GFP-Vamp7 localization pattern as it still forms rings around SGs. Insets show the outlined areas magnified (×2) and split into channels in panels (**a**,**b**). (**c**) Our proposed model for lysosomal fusions that regulate the maturation and crinophagic breakdown of SGs. Immature SGs (iSGs) bud from the trans-Golgi network (TGN), increase in size by homotypic fusions, then undergo a complex maturation process involving a series of lysosomal fusions. First, Arl8+ lysosomes fuse with the maturing SGs by the canonical Syx13–Snap29–Vamp7 SNARE complex, enabling subsequent fusions with the Lamp1 carrier vesicles, which in turn is mediated by the Syx13–Snap29–Ykt6 SNAREpin. These consecutive lysosomal fusions promote the progressive acidification and inner reorganization (decondensation) of SG contents. Mature SGs (mSGs) are eventually released by regulated exocytosis, while the residual SGs (rSGs) that evade secretion are selectively degraded by crinophagy, which requires further fusions between the endolysosomal compartment and rSGs.

We also wondered whether the two SNAREpins that mediate SG-lysosome fusions indeed function independently. Therefore, we investigated the localization of Vamp7 by using N-terminal GFP-tagged Vamp7 in the absence of the other R-SNARE, Ykt6. The loss of Ykt6 has not altered the localization pattern of Vamp7, as it is still able to form rings around larger SGs (Fig. 6a,b). This further suggests that the Ykt6- and Vamp7-containing SNAREpins independently regulate the maturation and crinophagic degradation of SGs by mediating fusion between SGs and Arl8+ (by Vamp7) or Arl8- but Lamp1+ (Vamp7, Ykt6) lysosome subpopulations.

## Discussion

In this work, we revealed that Ykt6 acts together with Syntaxin 13 and Snap29 to form a functional SNAREpin. This SNARE complex is required for efficient SG fusion with Lamp1 carrier vesicles, thereby promoting the maturation and crinophagic elimination of SGs. The first vesicle fusions occur just before the bulk secretion of SGs. These early fusions may drive the acidification and inner reorganization of SGs to promote SG maturation^17–19^. In line with this, SG-lysosome fusion is claimed to cause enhanced secretion in Trpml1^−/−^ mutant pancreatic acinar cells^20^. Our data indicate that the two R-SNAREs Ykt6 and Vamp7 play different roles in regulating the maturation of SGs, because SGs fail to acidify properly without Vamp7, while the silencing of *ykt6* did not prevent this.

Moreover, we found that Vamp7 is required for the localization multiple lysosomal markers to maturing glue granules, while Ykt6 only affects the fusion of SGs with Lamp1+ vesicles and it is dispensable for fusion with Arl8+ lysosomes. We hypothesize that maturing SGs first undergo Vamp7-mediated fusion with Arl8+ lysosomes, which is required for their maturation. The Ykt6-mediated fusions between SGs and Lamp1+ vesicles likely represent a later step of SG maturation. Although our findings suggest the existence of at least two separate vesicle subpopulations carrying these lysosomal markers (Arl8+ ones and Lamp1+/Arl8− ones), Arl8+ lysosomes possibly also contain Lamp1.

The coexistence and sequential contribution of multiple lysosomal subpopulations/Lamp1 carriers in distinct steps of SG maturation could be an advantage for secretory cells. Different vesicle subpopulations can act as carriers that deliver different lysosomal membrane proteins and enzymes that are required for lysosome biogenesis. The volume of SGs is enormous compared to these small vesicles, hence the desired concentration of lysosomal proteins in matured SGs or crinosomes could be fine-tuned by a series of membrane fusions with different lysosomal populations. This model is further supported by findings by others, showing that Vamp7 is required for the transport of lysosomal membrane proteins (LMPs, including Lamp1)^64^ or the potential role of Ykt6 in lysosomal enzyme transport^42,52^. Since we found that the vesicles to which Ykt6 localizes are positive only for Lamp1, but negative for Arl8 and the lysosomal protease Cathepsin L, Ykt6 appears to be required for the delivery of lysosomal membrane proteins such as Lamp1 itself to glue granules. As the highly glycosylated Lamp1 is essential for protecting the lysosomal membrane from acidic internal pH and enzymatic degradation^58^, the Ykt6-mediated delivery of these Lamp1 carrier vesicles to mature SGs could prepare them for the degradative crinosomal fate.

We have also demonstrated the importance of endosomal contribution to crinosome formation. PI3P+ endosomes are much smaller than SGs and initially form clusters among SGs before secretion^60–62^, eventually fusing with the residual, non-secreted mature SGs. One can assume that these endosomal fusions prepare the obsolete SGs for crinophagic breakdown, possibly through the recruitment of Rab7, which is implicated in crinophagic SG-lysosome fusion^3,5^. Importantly, we found that these fusions are equally hampered in the absence of either Ykt6 or Vamp7. Thus, the early Ykt6- and Vamp7-mediated vesicle fusions determine the subsequent fate and fusion potential of maturing SGs. Small PI3P+ endosomes that fuse with residual SGs are likely derived from endocytic activity that follows the programmed secretion of SGs. The convergence of secretory, endosomal, and even autophagic routes in lysosomes was also demonstrated in larval Drosophila fat tissue^65^.

Overall, our results refine the model of glue granule maturation and lysosome fusions: SGs probably first acquire the lysosomal small GTPase Arl8 and begin to acidify via fusion by the canonical Vamp7 containing SNAREpin. This primary fusion event engages maturing SGs for subsequent volume-increasing lysosomal fusions that already involve Lamp1+ lysosomes. Ykt6 reaches SGs by forming a SNAREpin with Syntaxin 13 Q_a_- and Snap29 Q_bc_-SNAREs to mediate SG-Lamp1 carrier vesicle fusion, and these separate fusion events together promote the maturation and crinophagic degradation of residual glue granules after secretion (Fig. 6c).

## Methods

### Drosophila genetics

Fly stocks were maintained on standard yeast-cornmeal-agar medium at 25 °C temperature. To avoid undesirable effects of Ykt6 on SG biogenesis, temperature sensitive tubP-Gal80 construct was used to temporally control the expression of ykt6 RNAi transgenes^53^. These crosses were shifted from the 18 °C restrictive temperature to 29 °C for 36 h at the late (wandering) L3 stage. Accordingly, separate controls were used for room temperature vamp7 and temperature-induced ykt6 RNAi experiments. The w^1118^ (#3605), fkh-Gal4 (#78060), tubP-Gal80^ts^ (#7017 and #7108), Sgs3-GFP (#5884) and the UAS-GFP-myc-2xFYVE (#42712) lines were obtained from Bloomington Drosophila Stock Center. The UAS-Ykt6^NIG.1515R^ (#1515R-1) (ykt6 RNAi/1 in the text) RNAi line was obtained from NIG-Fly (National Institute of Genetics)^35^. The UAS-Ykt6^KK101343^ (#v105648) (ykt6 RNAi/2 in the text) and UAS-Vamp7^KK107576^ (#v108733) RNAi stocks were purchased from Vienna Drosophila Resource Center. The Sgs3-dsRed line was kindly provided by Andrew Andres (University of Nevada, US). Sgs3-GFP; fkh-Gal4, the Sgs3-dsRed; fkh-Gal4, the Sgs3-dsRed, UAS-GFP-myc-2xFYVE; fkh-Gal4 and the Sgs3-dsRed, Sgs3-GFP; fkh-Gal4 lines were used to study the endo-lysosomal transport to secretory granules or secretory granule acidification. For our experiments we used late L3 staged larvae that had already completed their wandering (considered as 2 h before puparium formation (2 h bpf)) or white prepupae (pp).

### LysoTracker Red (LTR) staining

The larval salivary glands were dissected in cold PBS (pH 7.4) and permeabilized for 30 s (2 h bpf) or 15 s (pp) in 0.05% Triton X-100-PBS (PBTX) solution. The samples were rinsed in PBS (3 × 30 s) and incubated for 2 min in 0.5 nM LTR (in PBS, Invitrogen) staining solution, then washed in PBS and mounted with 9:1 PBS: glycerol solution that contains 1 µg/ml DAPI (4′,6-diamidino-2-phenylindole, Sigma Aldrich) to stain the nuclei of cells.

### Immunohistochemistry

The larval salivary glands were dissected in cold PBS, gently permeabilized with 0.05% PBTX solution either for 30 s (2 h bpf) or 15 s (prepupae) and fixed in 4% formaldehyde-PBS (40 min, RT). Then, the samples were rinsed with PBS (3 × 5 min, RT), incubated in a blocking solution (5% fetal calf serum in 0.1% PBTX, 30 min, RT), and incubated with the first antibodies dissolved in the blocking solution (ON, 4 °C). After washing (3 × 15 min PBTX), salivary glands were incubated in blocking solution (30 min, RT), then with the secondary antibodies diluted in blocking solution (3 h, RT). Thereafter samples were incubated in 4% NaCl solution (15 min, RT) that was supplemented with Hoechst (1:200, Sigma-Aldrich) nuclear dye and washed (2 × 15 min in 0.1% PBTX, 3 × 15 min in PBS). The specimens were dissected and mounted in Vectashield (Vector Laboratories).

For the salivary gland immunostainings rabbit anti-Arl8 (1:100, DSHB)^21^, rabbit anti-dLamp1 (1:1000, kind gift of Andreas Jenny)^58^, rat anti-Ykt6 (1:30)^35^, rat anti-mCherry (1:300) and rabbit anti-CathL/MEP (1:100, Abcam, #ab58991)^31,35^ primary and the AlexaFluor488-conjugated anti-mouse, anti-rabbit, anti-goat and AlexaFluor568-conjugated anti-mouse, anti-rabbit and anti-rat secondary antibodies (all 1:1000, Invitrogen) were used.

### Fluorescent imaging

Fluorescent images were taken at room temperature with an AxioImager M2 microscope (Zeiss) equipped with an ApoTome.2 structured illumination unit, Orca-Flash 4.0 LT3 digital sCMOS camera (Hamamatsu Photonics), EC Plan-Neofluar 20×/0.50, Plan-Apochromat 40×/0.95 and Plan-Apochromat 63×/1.4 Oil objectives (Zeiss). Raw images were processed with ZEN2.3 lite Microscopy Software and Photoshop CS4 (Adobe Systems). To improve clarity in Figs. 2, 3, 4 and 6 consecutive optical slices spanning a depth of 3 µm were projected onto single images. Single focal planes were presented in other figures, including colocalization tests in Figs. 1 and 5.

### GST pulldown assay

SNARE fragments were cloned into pETARA or/and pETMBP vectors, which contain C-terminal Glutathione S-transferase/Maltose Binding Protein tag and C-terminal hexahistidine-tag, respectively, using BamHI and XhoI restriction sites. Syx13 was amplified from the EST LD27581 (DGRC Stock 4205; https://dgrc.bio.indiana.edu//stock/4205; RRID:DGRC_4205) with primers 5′-ATCGGATCCCACGACATGCTCGAC-3′ and 5′-ATCCTCGAGCGCCTTGGCCAGTTC-3′. The remaining constructs were already reported in an earlier study^35^.

For pulldown experiments, recombinant SNARE constructs were expressed overnight at 18 °C in E. coli Rosetta(DE3) pLysS (Novagen) cells induced with 0.1 mM IPTG at OD 0.6–0.7. Cells were then centrifuged and suspended in lysis buffer (pH 8.0, 50 mM Na_2_HPO_4_, 300 mM NaCl, 20 mM imidazole, 0.1% Triton-X, 5 mM-β-mercaptoethanol, protease inhibitors). Lysed samples were centrifuged (48.000*g*, 30 min). Ni–NTA resin was added to the supernatant and incubated for 30 min at 4 °C. Beads were washed with washing buffer (pH 8.0, 50 mM Na_2_HPO_4_, 1 M NaCl, 40 mM imidazole, 0.1% Triton-X, 5 mM β-mercaptoethanol) and then eluated in Elution buffer (pH 8.0, 20 mM Tris, 200 mM NaCl, 400 mM imidazole, 10% glicerol, 0.1% Triton-X, 5 mM-β-mercaptoethanol) used for pulldown assays. Prey proteins for pulldown experiments were purified with further MBP affinity chromatography using standard protocols. All resins were from GE Healthcare.

For GST pulldown assays, the glutathione resin (New England BioLabs) was first equilibrated with binding buffer (20 mM Tris, 50 mM NaCl, 0.1% Triton-X, 2 mM β-β-mercaptoethanol), then 0.5 mg GST fused SNARE proteins (and GST as negative control) were immobilized on it. In the binding experiments, 40 μl of resin saturated with baits were incubated in the presence of 20 μM preys in binding buffer (200 μl total volume, 30 min at 4 °C). Glutathione beads were pelleted with centrifugation (200 g, 2 min) and washed 3× with 20 mM Tris, 300 mM NaCl, 0.1% Triton-X, 2 mM β-mercaptoethanol. Retained proteins were eluted from the resin with an SDS loading buffer. Samples were subjected to SDS-PAGE and interactions were detected by Coomassie protein dye. The original gel image is provided in the Supplementary Information (Supplementary Fig. S1).

### Statistics

ImageJ software (National Institutes of Health, Bethesda, Maryland, US) was used for quantitative analysis of fluorescent structures. Overlap of the markers was assessed by Pearson’s correlation analysis using the Coloc2 plugin (Fig. 1) or in the case of membrane markers encircling granular structures (Fig. 5), the signal was calculated manually. For manual colocalization assessment, 200 immunolabeled structures were selected. The threshold for LTR quantification was set by the same person in a dark room in all images and structures were counted. The structure diameter range was set to 1–99 µm^2^ to exclude background noise and unrealistic clumped structures of several SG sizes (Fig. 2). The ring-like fluorescent structures in Figs. 3 and 4 were selected manually by the same person and the percentage of them located around SGs was examined. For pairwise comparisons of datasets that followed Gaussian distribution unpaired t-test (Figs. 3i,j, 4f,g) or where at least one of the datasets followed non-Gaussian distribution, Mann–Whitney U test (Figs. 2, 3k,l) were performed. To analyze multiple datasets with Gaussian distribution, one-way ANOVA with Tukey’s post hoc test was performed (Fig. 1d). The distribution tests of datasets and statistical analyses were carried out using GraphPadPrism 9.0.0 software (Boston, Massachusetts, US). All source data about the quantifications related to the presented experiments are available as Supplementary Information (Supplementary Table S1).

### Supplementary Information

Supplementary Figure S1.

Supplementary Table S1.

## Data Availability

All data needed to evaluate the conclusions in this paper are present in the paper and its Supplementary Information. Other data associated with the article, such as raw data, are available upon request.

## Materials availability

All materials, Drosophila stocks and related information are available from the corresponding authors upon reasonable request.
