# Supplementary material for: The Ykt6–Snap29–Syx13 SNARE complex promotes crinophagy via secretory granule fusion with Lamp1 carrier vesicles
Source: Sci Rep. 2024 Feb 8;14:3200. doi: 10.1038/s41598-024-53607-x (PMC10853563; doi:10.1038/s41598-024-53607-x)
Supplement: Supplementary file 1 — Supplementary Figure S1. [file 41598_2024_53607_MOESM1_ESM.pdf]

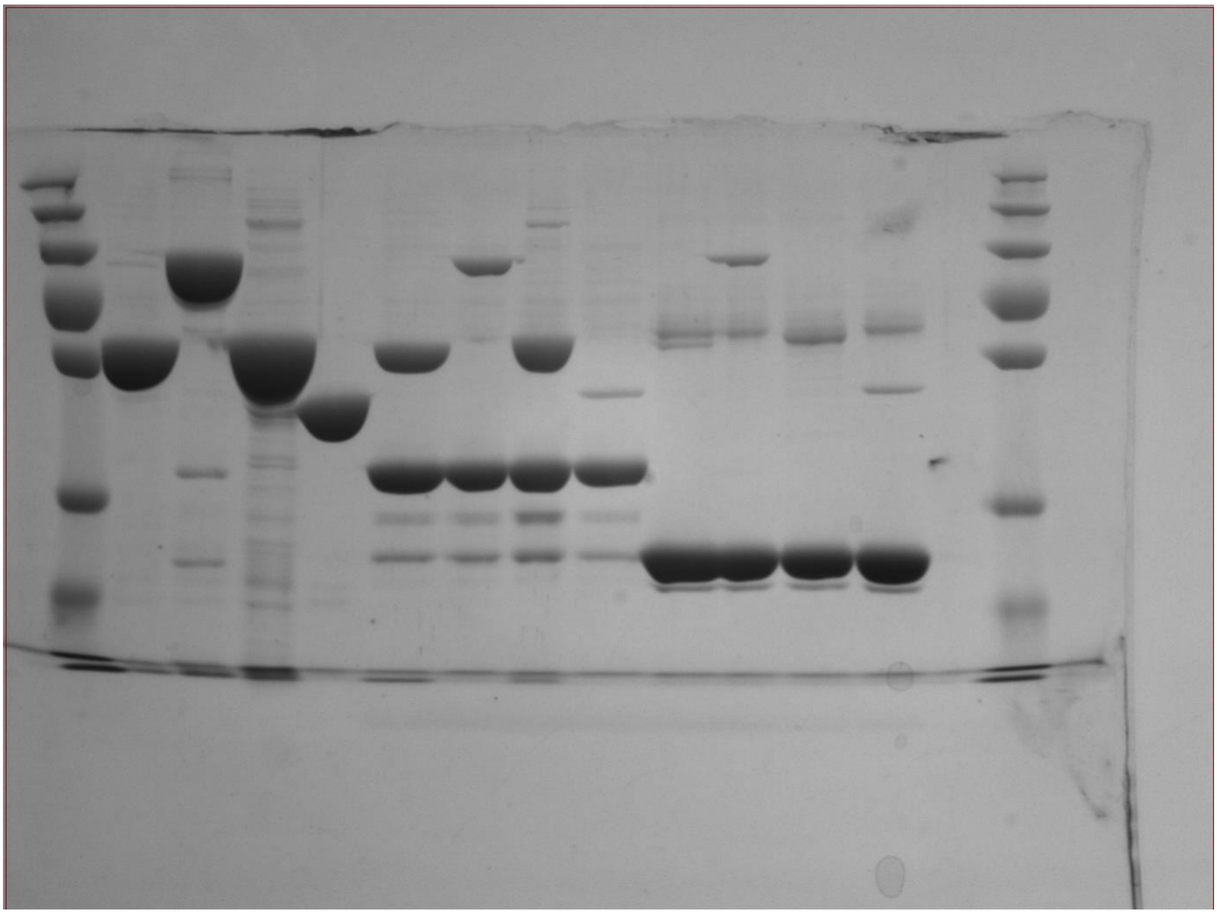

Supplementary Figure S1. **The original gel image of the GST-pulldown experiment presented in Fig. 1e.**
